# Nematode Community Profiling in Organic and Conventional Spinach Systems

**DOI:** 10.1002/pei3.70201

**Published:** 2026-08-04

**Authors:** Scolasticah K. Munene, Hannah Karuri, Mariciano Mutiga

**Affiliations:** ^1^ Department of Biological Sciences University of Embu Embu Kenya; ^2^ Department of Dryland Agriculture and Natural Resources Management Tharaka University Marimanti, Meru Kenya

**Keywords:** organic amendments, soil biodiversity, soil health, sustainable agriculture

## Abstract

Agricultural management practices play a significant role in shaping soil biological communities and ecosystem processes. Soil nematodes are valuable biological indicators of soil health because they occupy different trophic levels and are sensitive to alterations in environmental conditions. This study assessed the influence of organic and conventional farming systems on soil nematode communities in spinach production systems in Embu County, Kenya. We hypothesized that organic farming systems would support higher nematode diversity and a more complex community structure than conventional systems. Soil samples were collected from farms under the two management systems and analyzed using high‐throughput sequencing targeting the 18S rRNA gene. A total of 394 amplicon sequence variants (ASVs) representing 73 nematode genera were identified across the two systems. Numerical differences were observed in the abundance of nematode families, genera, and trophic groups between the two farming systems. Cephalobidae, Prismatolaimidae, and Trichodoridae showed higher relative abundance in the conventional system, while Rhabditidae, Monhysteridae, and Qudsianematidae were relatively more abundant in the organic system, but these differences were not statistically significant. *Acrobeloides*, *Prismatolaimus*, and *Paratrichodorus* were relatively more abundant in the conventional system, whereas *Eumonhystera*, *Aphelenchoides*, *Diploscapter*, and *Microdorylaimus* were more abundant in organic farms; however, these differences were not significant (*p* > 0.05). Similarly, fungivores and plant‐parasitic nematodes were relatively more abundant in conventional farms, whereas bacterivores, predators, and omnivores were more abundant in organic farms, although these differences were also not statistically significant (*p* > 0.05). No significant differences were observed in alpha or beta diversity between the two farming systems. Our work establishes a valuable baseline for future studies aimed at enhancing the sustainable management of spinach agroecosystems.

## 1 Introduction

Spinach ( *Spinacia oleracea* ), a leafy vegetable of significant nutritional and economic importance (Bhattarai and Shi 2021), is cultivated worldwide under both organic and conventional systems (Bao et al. 2023). Spinach production in some parts of Africa is primarily characterized by small‐scale cultivation, on limited land, under rain‐fed or irrigated conditions, often integrated into mixed cropping systems or intensive vegetable production units (Mdoda et al. 2022). Management practices vary from low‐input organic systems, which utilize farmyard manure, compost, and non‐chemical pest control strategies, to more intensive conventional systems that use synthetic fertilizers and pesticides to enhance productivity and meet market requirements (Betek and Jumbam 2015; Mdoda et al. 2022; Mwinga et al. 2022). These contrasting production systems, together with their interactions with soil properties, significantly affect soil health and below‐ground microorganisms, including nematodes (Ugarte et al. 2013; Yang et al. 2021).

In Kenya, spinach is a vital part of the horticultural industry, significantly supporting smallholder farmers' livelihoods and the urban food supply. Recent data shows that in 2024, Kenya produced approximately 242,720 t of spinach (FAOSTAT 2025). Smallholder farmers in Kenya play a major role in vegetable production, with surveys indicating that approximately 31% of some households producing crops grow spinach alongside other essential horticultural crops such as kale and cabbage (Kilelu et al. 2024). Spinach is popular due to its quick growth cycle and the ability to harvest it multiple times in a season, ensuring a steady supply to local markets and regular income. The crop's minimal space and input needs make it ideal for farms smaller than one acre, especially in peri‐urban areas, where horticulture meets both commercial and subsistence demands (Ogendi et al. 2019).

Spinach production in Kenya is affected by several constraints, including pests and diseases (Okoma et al. 2025). Plant‐parasitic nematodes (PPN) are a major threat to vegetable production in smallholder farming systems in Kenya (Chitambo et al. 2019). Diversity and abundance of PPN are influenced by farm management practices (Garcia et al. 2018). It is therefore important for smallholder farmers to use practices that reduce PPN populations and promote beneficial soil organisms, thereby enhancing soil health and the soil's natural pest‐suppressive ability (Sánchez‐Moreno and Ferris 2018). Agricultural management greatly influences soil organisms, and differences between organic and conventional systems result in changes in nematode community composition (Briar et al. 2016). Through the application of organic manure and minimal use of pesticides, organic farming aims at sustainable food production. Long term use of organic farming system results in increased soil organic carbon (Gattinger et al. 2012), suppression of above‐ground pests, and increased soil biodiversity, hence increasing free‐living nematode populations (Yang et al. 2021).

Free‐living nematodes such as bacterivores and fungivores are beneficial because of their contribution to nutrient cycling and organic matter decomposition within the soil food web, which is important for crop production (Sánchez‐Moreno and Ferris 2018). In contrast, conventional farming practices often rely on synthetic fertilizers and chemical pesticides, which can negatively impact soil microbial diversity and beneficial nematodes (Ilieva‐Makulec et al. 2016), leading to a decline in ecological stability and biological control capacity (Lupatini et al. 2017). While chemical inputs are effective in controlling parasitic nematodes temporarily, they can reduce the populations of non‐target beneficial nematodes, disrupting food web dynamics that are critical for soil health (Tahat et al. 2020). Sustainable agricultural practices that promote the presence of beneficial nematodes and exploit their ecological roles offer promising alternatives to chemical control. Understanding the influence of conventional and organic farming practices on nematode assemblages is therefore essential for developing sustainable crop management strategies. Here, we present a comparative analysis of the diversity and abundance of nematode communities in organically and conventionally managed spinach farms in Embu County, Kenya. We used 18S rRNA metabarcoding, a high‐throughput sequencing (HTS) approach that enables comprehensive detection of nematode taxa (Hayden et al. 2025). This approach allows more accurate assessment of nematode diversity and ecological patterns (Kenmotsu et al. 2021). However, HTS‐based nematode studies remain scarce in African agroecosystems, where most research is still based on conventional morphological identification. The results will contribute to a better understanding of the influence of farming practices on nematode communities in spinach agroecosystems and also inform the development of agricultural practices that promote ecological balance and support sustainable spinach production.

## 2 Materials and Methods

### 2.1 Study Site and Sampling

This study was conducted on smallholder spinach farms managed under organic and conventional production systems in Embu County, Kenya (−0.531323, 37.466874). The region falls within the Upper Eastern highlands and experiences two rainfall seasons annually. The mean annual rainfall and temperature in Embu range from 1000 to 1600 mm and 20.6°C and 22.9°C, respectively. Soils in the area are predominantly nitisols, supporting intensive vegetable production. To ensure comparability between systems, farms were selected based on consistent management history, similar cropping stage (6 weeks after planting), and comparable soil type. Conventional farms were managed using commercial NPK fertilizers alongside synthetic pesticides such as cypermethrin, while organic farms primarily relied on cow manure as a nutrient source. Both systems were maintained under their respective management regimes for 5 years. Five organic (SF1, SF2, SF3, SF4, and SF05) and five conventional (SM1, SM2, SM3, SM4, and SM05) locations were sampled in September 2025, with a minimum distance of 3 km between locations. At each location, soil samples were collected from five farms. Within each farm, a representative 30 m × 30 m sampling area was established. Soil cores were collected from the top 10 cm layer using a sterilized 5 cm diameter auger after removing large surface debris. A total of 30 sampling points were established within each sampling area using three W‐shaped transects, and the collected soil cores were pooled to generate one composite sample per farm (Neilson et al. 2026; Wiesel et al. 2015). The five farm‐level composite samples from each location were then pooled to produce a single composite sample representing that location. Soil samples for molecular analysis were immediately stored at −80°C for 2 days prior to DNA extraction. A separate 1 kg sub‐sample was used for analysis of Nitrogen (Bremner and Mulvaney 1982), Carbon (Anderson and Ingram 1993), Phosphorous (Mehlich et al. 1962), sand, silt, and clay (Klute 1986) at the Kenya Agricultural and Livestock Research Organization, National Agricultural Research Laboratories.

### 2.2 DNA Extraction and PCR Amplification

DNA was extracted from 250 mg (Ali et al. 2025) of homogenized soil using a DNeasy PowerSoil Pro Kit (Qiagen, Germany) following the manufacturer's instructions. Extraction blanks were included as negative controls. The 18S rRNA gene was amplified using nematode primer pair NF1 (forward: GGTGGTGCATGGCCGTTCTTAGTT) and 18Sr2b (reverse: TACAAAGGGCAGGGACGTAAT) (Porazinska et al. 2009). PCR amplification reactions were prepared in 25 μL volumes following the conditions described by Resch et al. (2022). Initial denaturation was performed at 95°C for 2 min, followed by 35 cycles consisting of denaturation at 95°C for 40 s, annealing at 58°C for 40 s, and extension at 72°C for 1 min. A final extension at 72°C for 10 min was then carried out. Amplified PCR products were purified before library preparation. Paired‐end sequencing was carried out on an Illumina NovaSeq 6000 (2 × 250 bp; Illumina Inc., CA, USA) at Molecular Research Laboratory (Shallowater, TX, USA).

### 2.3 Bioinformatics and Statistical Analysis

Based on the protocol by Bolyen et al. (2019), raw paired‐end FASTQ files were analyzed using QIIME 2 and the cutadapt plugin (Martin 2011) was used to remove the primer sequences. The forward and reverse reads were truncated at 200 and 220 bp, respectively, based on a quality score threshold of Q37, and merged successfully after quality filtering. DADA2 was used for denoising, quality filtering, paired‐end merging, chimera detection and amplicon sequence variants (ASVs) generation (Callahan et al. 2016). The SILVA (Quast et al. 2012) and 18S‐NemaBase (Gattoni et al. 2023) databases were used for taxonomy assignment. The ASVs tables and associated data were imported to R for subsequent analysis. Raw sequence data are deposited in the NCBI Sequence Read Archive under PRJNA1426156. Nematode ASVs were classified according to genus and trophic group (bacterivores, omnivores, fungivores, predators, plant parasites) following Yeates et al. (1993) and Nemaplex (http://nemaplex.ucdavis.edu). Genera were further categorized into colonizer‐persister (c‐p) scales from 1 (opportunistic) to 5 (disturbance‐sensitive) for free‐living nematodes and p–p scales for plant parasites (Bongers 1990; Bongers and Bongers 1998). The nematode‐based indices (Maturity Index; MI, Maturity Index 2–5; MI 2–5, Structure Index; SI, Enrichment Index; EI, Plant‐parasitic Index; PPI, Basal Index; BI and Channel Index; CI) were computed using the online software Nematode Indicator Joint Analysis tool (Sieriebriennikov et al. 2014). Normality (Shapiro–Wilk test) and homoscedasticity (Levene's test) were assessed for all data. Relative abundance differences across groups were then compared using one‐way ANOVA for normally distributed data or Kruskal–Wallis tests for violations, with *p* values adjusted using the Benjamini‐Hochberg false discovery rate (FDR) correction. The number of observed ASVs, Faith's PD, beta diversity, Shannon, and Simpson indices were calculated on rarefied data. One way ANOVA was used to determine the differences in the diversity indices that were normally distributed, while data that did not meet this requirement were analyzed using Wilcoxon test with *p* values adjusted via Benjamini‐Hochberg FDR correction. For beta diversity analysis, Bray–Curtis dissimilarity matrices were compared between sites using PERMANOVA (9999 permutations) and visualized through Principal Coordinates Analysis (PCoA). Redundancy analysis (RDA) was used to determine the relationship between the abundance of nematodes and soil properties. Permutation‐based ANOVA was used to assess the significance of the model (nperm = 999).

## 3 Results

**FIGURE 1:**
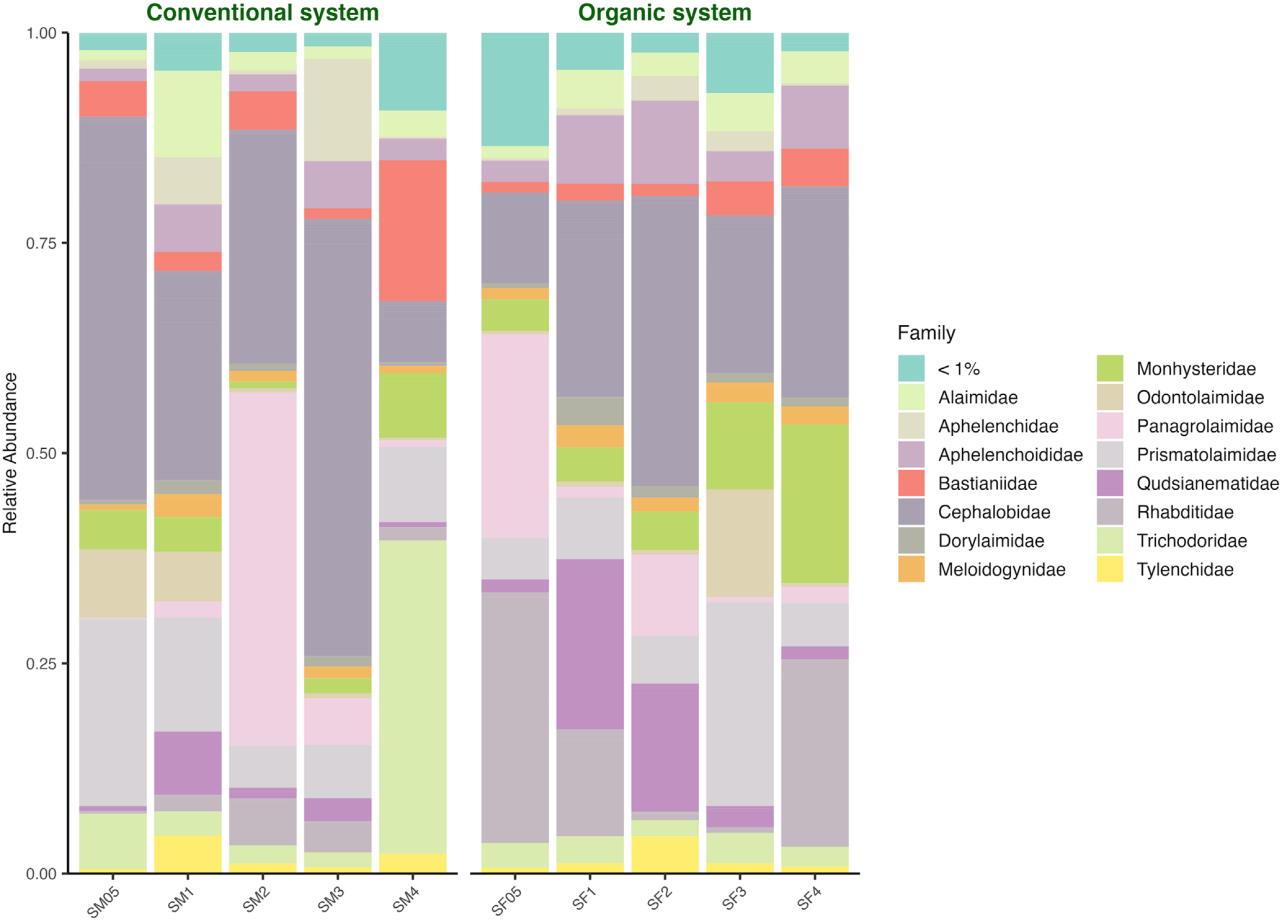
Top 15 nematode families in organic and conventional spinach cropping systems in Embu, Kenya. SF1–SF05 represent organic locations, while SM1–SM05 represent conventional locations.

**FIGURE 2:**
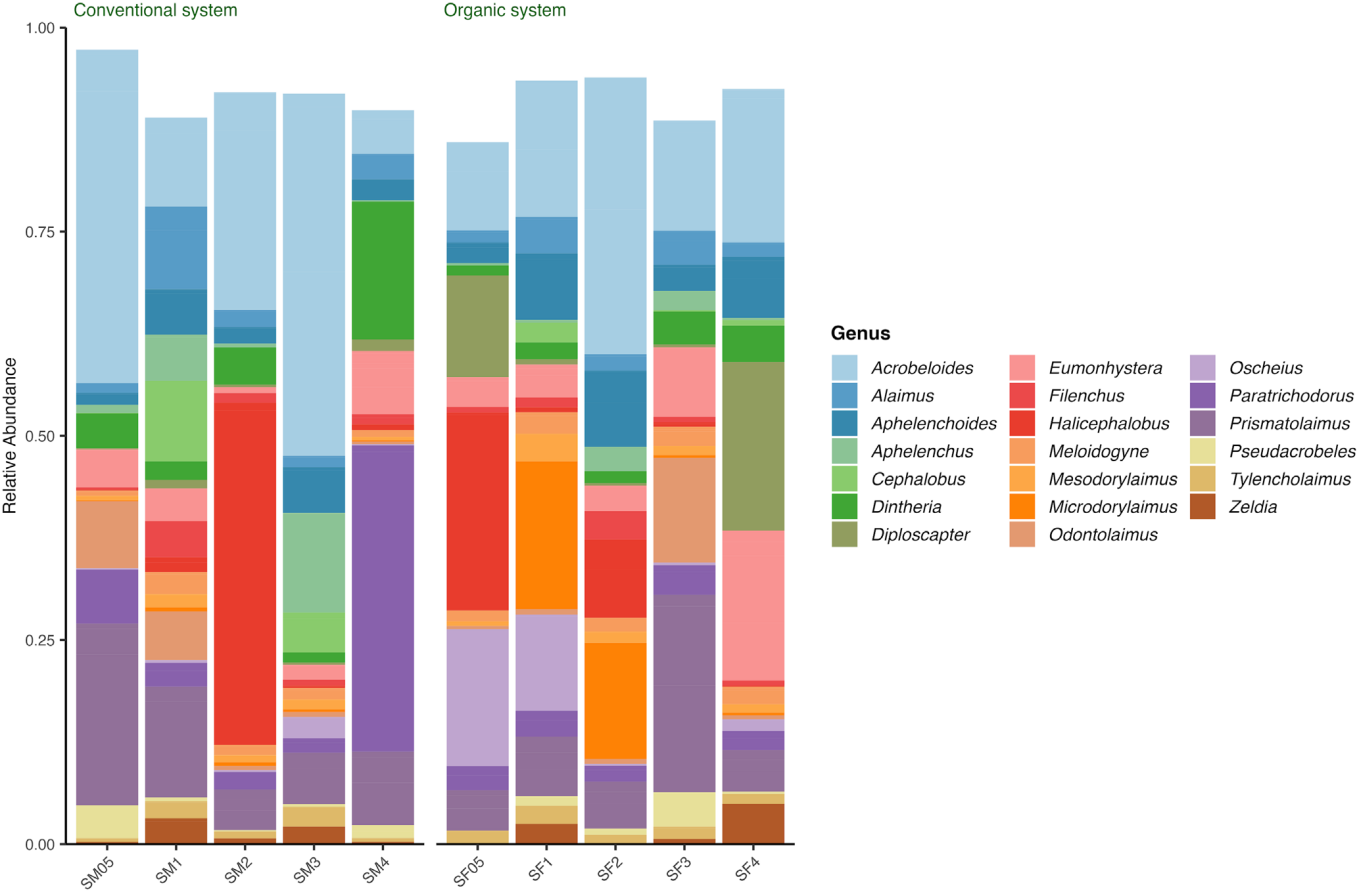
Top 20 nematode genera in organic and conventional spinach cropping systems in Embu, Kenya. SF1–SF05 represent organic locations, while SM1–SM05 represent conventional locations.

**FIGURE 3:**
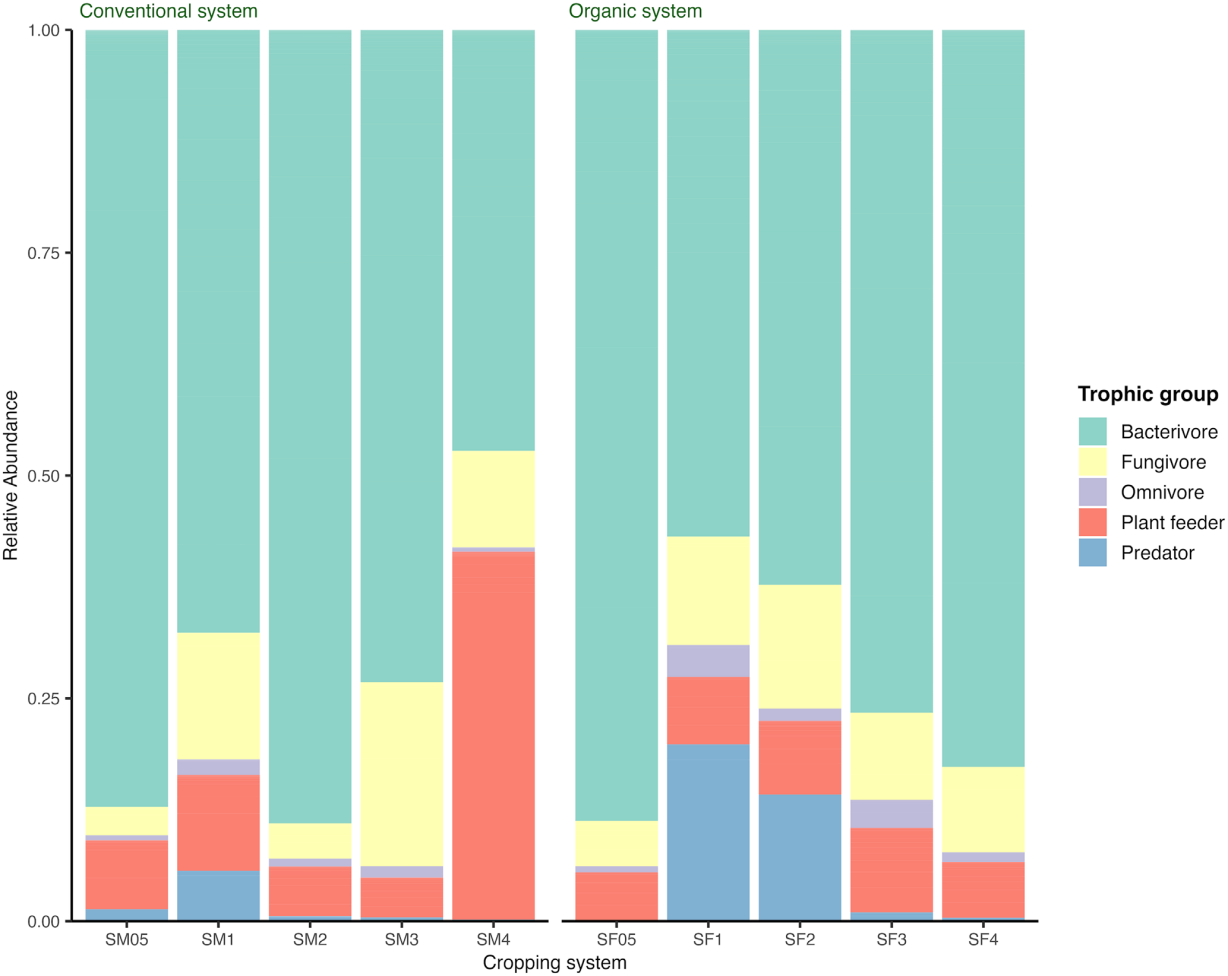
Nematode trophic groups in organic and conventional spinach cropping systems in Embu, Kenya. SF1–SF05 represent organic locations, while SM1–SM05 represent conventional locations.

In the organic and conventional cropping systems, a total of 266,731 reads were retained after quality filtering. The mean number of reads per sample was 39,320 ± 17,794 in the conventional system and 14,026 ± 5744 in the organic system. There were 394 ASVs representing 73 nematode genera (Table 1; Figure S1). Numerical differences were observed in the abundance of nematode families, genera, and trophic groups between the two farming systems. Cephalobidae (31.5%), Prismatolaimidae (11.9%), and Trichodoridae (10.1%) were more abundant in the conventional system, whereas Rhabditidae (13.3%), Monhysteridae (8.3%), and Qudsianematidae (8.2%) were more abundant in the organic system; however, these differences were not statistically significant (*p* > 0.05; Figure 1). At the genus level, we observed numerically higher but not significant (*p* > 0.05) abundances of *Acrobeloides* (25.6%), *Prismatolaimus* (11.2%), and *Paratrichodorus* (10.2%) in the conventional system. Organic farms, on the other hand, had a numerically higher but not significant (*p* > 0.05) abundance of *Eumonhystera* (7.5%), *Aphelenchoides* (6.2%), *Diploscapter* (6.9%), and *Microdorylaimus* (6.6%) (Figure 2). The relative abundance of fungivores (10.5%) and plant parasites (14.0%) was numerically higher (*p* > 0.05) in conventional farms. Organic fields had a greater abundance (*p* > 0.05) of bacterivores (73.4%), predators (7.1%), and omnivores (2.0%) (Figure 3), and these differences were not significant.

**TABLE 1:** Nematode genera, C‐p/P–p class and feeding type in organic and conventional spinach cropping systems in Embu, Kenya based on Nematode Indicator Joint Analysis (NINJA) tool.

Genus	C‐p/P–p class	Feeding type
*Axonchium*	5	Herbivores—ectoparasites
*Boleodorus*	2	Herbivores—epidermal/root hair feeders
*Cactodera*	3	Herbivores—sedentary parasites
*Coslenchus*	2	Herbivores—epidermal/root hair feeders
*Criconema*	3	Herbivores—ectoparasites
*Dorylaimellus*	5	Herbivores—ectoparasites
*Ecphyadophora*	2	Herbivores—epidermal/root hair feeders
*Helicotylenchus*	3	Herbivores—semi‐endoparasites
*Labrys*	2	Herbivores—epidermal/root hair feeders
*Meloidogyne*	3	Herbivores—sedentary parasites
*Paratrichodorus*	4	Herbivores—ectoparasites
*Paratylenchus*	2	Herbivores—ectoparasites
*Pratylenchus*	3	Herbivores—migratory endoparasites
*Rotylenchulus*	3	Herbivores—sedentary parasites
*Schistonchus*	2	Herbivores—migratory endoparasites
*Xiphinema*	5	Herbivores—ectoparasites
*Aphelenchoides*	2	Fungivores
*Aphelenchus*	2	Fungivores
*Diphtherophora*	3	Fungivores
*Ditylenchus*	2	Fungivores
*Filenchus*	2	Fungivores
*Pseudhalenchus*	2	Fungivores
*Tylencholaimus*	4	Fungivores
*Tylolaimophorus*	3	Fungivores
*Acrobeles*	2	Bacterivores
*Acrobeloides*	2	Bacterivores
*Alaimus*	4	Bacterivores
*Bunonema*	1	Bacterivores
*Cephalobus*	2	Bacterivores
*Ceratoplectus*	2	Bacterivores
*Chiloplacus*	2	Bacterivores
*Cruznema*	1	Bacterivores
*Demaniella*	1	Bacterivores
*Dintheria*	3	Bacterivores
*Diplogastrellus*	1	Bacterivores
*Diploscapter*	1	Bacterivores
*Drilocephalobus*	2	Bacterivores
*Eucephalobus*	2	Bacterivores
*Eumonhystera*	2	Bacterivores
*Geomonhystera*	2	Bacterivores
*Halicephalobus*	1	Bacterivores
*Isolaimium*	5	Bacterivores
*Mesorhabditis*	1	Bacterivores
*Odontolaimus*	3	Bacterivores
*Oscheius*	1	Bacterivores
*Panagrolaimus*	1	Bacterivores
*Paramphidelus*	4	Bacterivores
*Poikilolaimus*	1	Bacterivores
*Prismatolaimus*	3	Bacterivores
*Procephalobus*	1	Bacterivores
*Prodesmodora*	3	Bacterivores
*Protorhabditis*	1	Bacterivores
*Pseudacrobeles*	2	Bacterivores
*Rhabditis*	1	Bacterivores
*Rhabdolaimus*	3	Bacterivores
*Zeldia*	2	Bacterivores
*Discolaimus*	4	Predators
*Ecumenicus*	4	Predators
*Ironus*	4	Predators
*Labronema*	4	Predators
*Microdorylaimus*	4	Predators
*Mononchus*	4	Predators
*Paractinolaimus*	5	Predators
*Pristionchus*	1	Predators
*Seinura*	2	Predators
*Tripyla*	3	Predators
*Tripylella*	3	Predators
*Amblydorylaimus*	5	Omnivores
*Aporcelaimellus*	5	Omnivores
*Enchodelus*	4	Omnivores
*Laimydorus*	4	Omnivores
*Mesodorylaimus*	4	Omnivores
*Rhyssocolpus*	4	Omnivores

**FIGURE 4:**
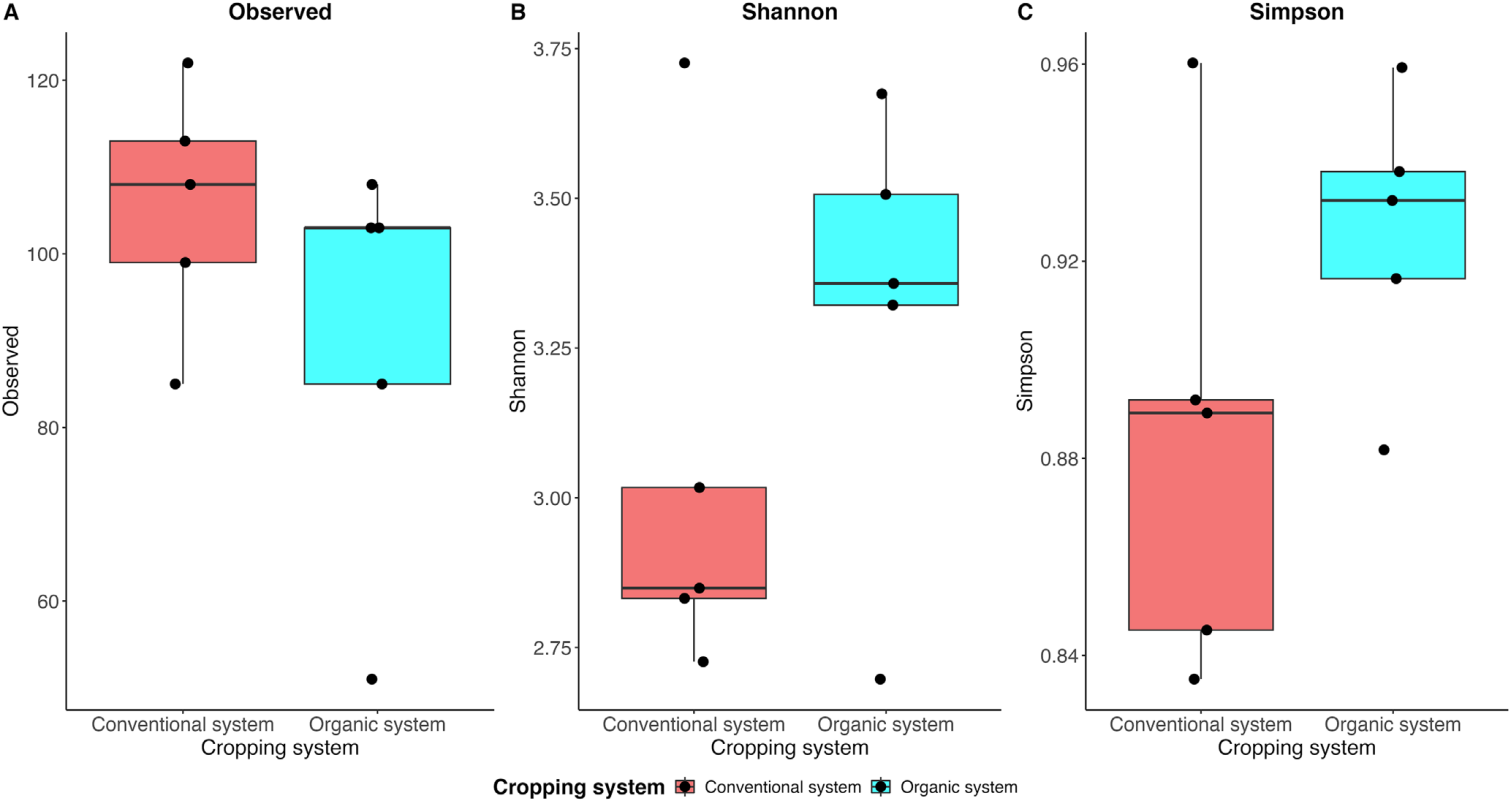
Observed ASVs, Shannon and Simpson diversity indices of nematode communities in organic and conventional spinach cropping systems in Embu, Kenya. No statistically significant differences were observed across the diversity indices (*p* > 0.05).

**FIGURE 5:**
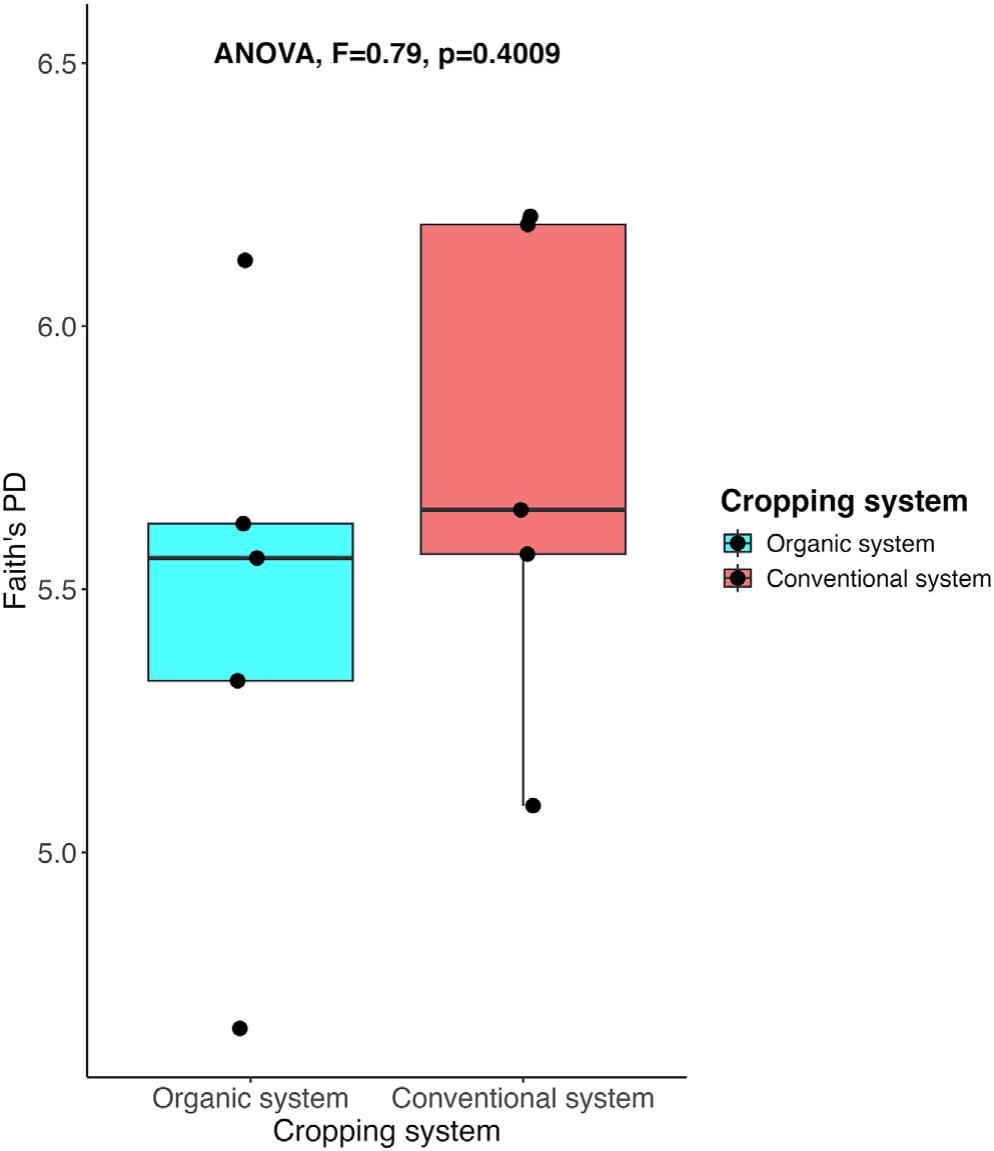
Faith's Phylogenetic diversity of nematode communities in organic and conventional spinach cropping systems in Embu, Kenya.

**FIGURE 6:**
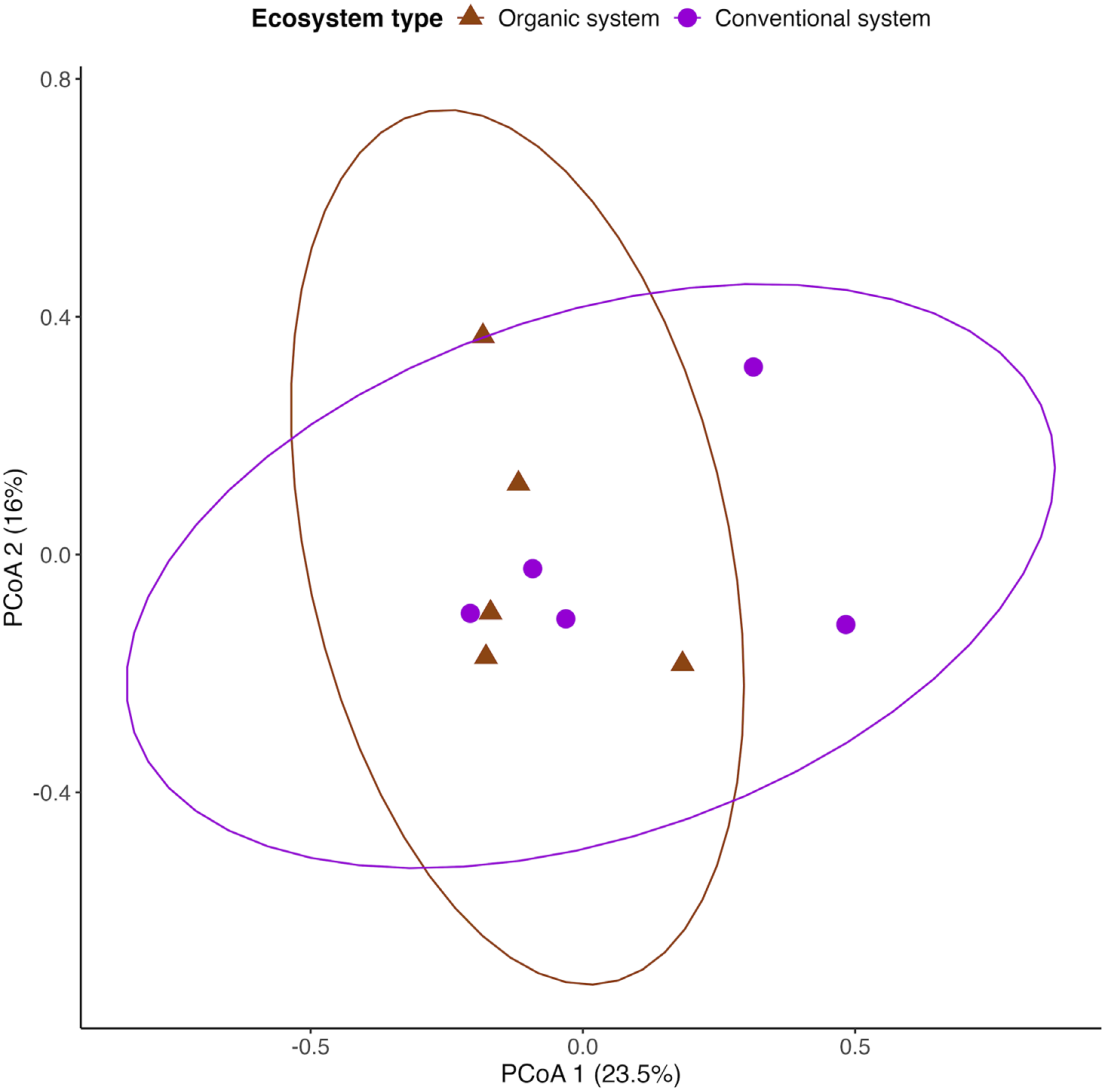
Principal coordinate analysis (PCoA) of nematode communities in organic and conventional spinach cropping systems in Embu, Kenya. The first two axes explain 39.5% of the variation in community composition. PERMANOVA indicated no significant differences between groups (*R* ^2^ = 0.11, *p* = 0.463).

**FIGURE 7:**
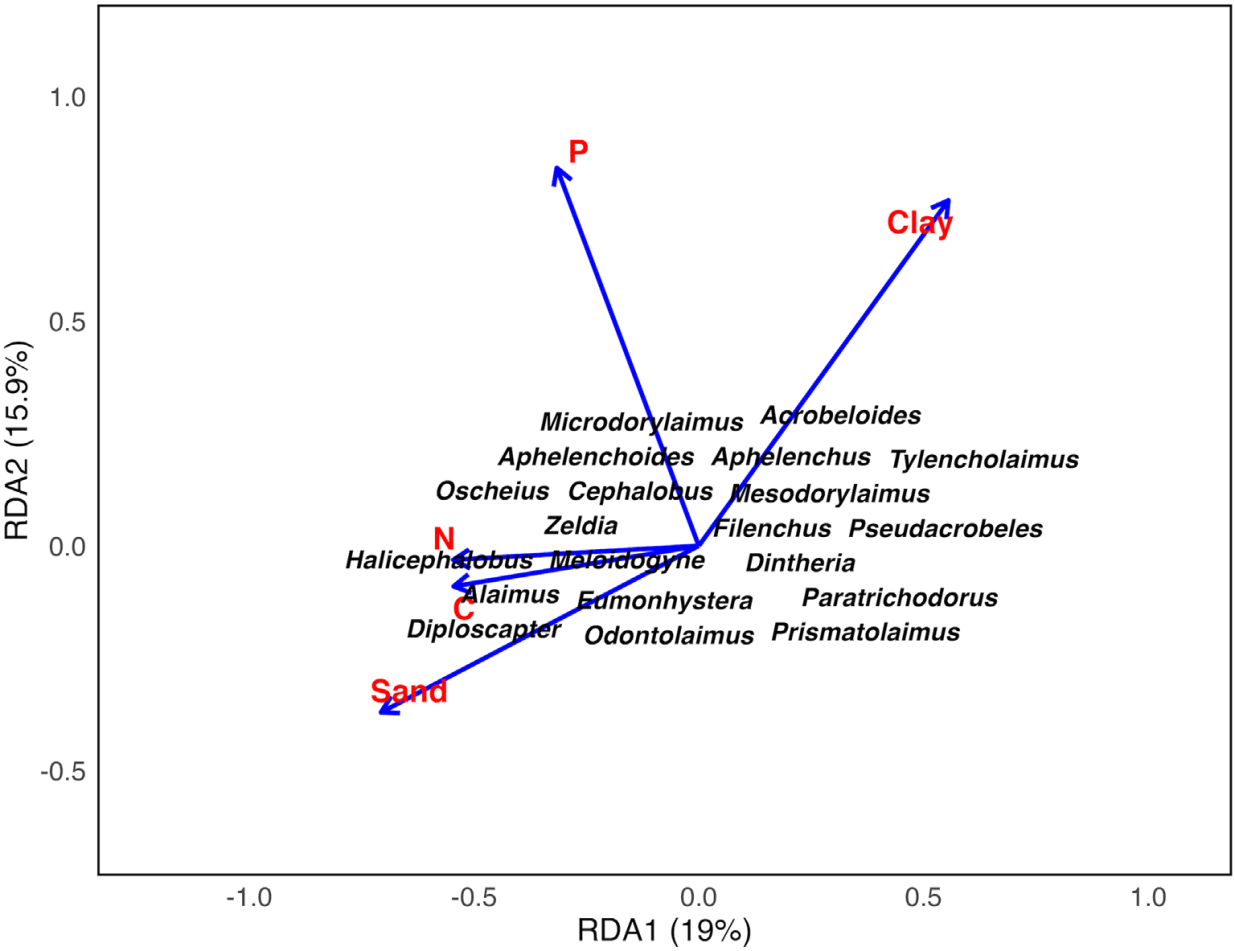
Redundancy analysis of nematode communities and soil variables in organic and conventional spinach cropping systems in Embu, Kenya. The RDA model was not statistically significant (*p* > 0.05).

The PPI and CI were higher in the conventional system, while the BI, EI, and SI were lower compared to the organic system; however, these differences were not significant (*p* > 0.05; Table 2). Analysis of alpha diversity showed higher values for Shannon and Simpson indices in organic systems, but the differences were not significant. Similarly, observed ASVs were higher in conventional fields, but these differences were not significant (*p* > 0.05; Figure 4). There was no significant difference (*p* > 0.05) in Faith's phylogenetic diversity (Figure 5). Beta diversity analysis showed an overlap in nematode community composition between organic and conventional systems, with no significant differences detected (PERMANOVA, *R* ^2^ = 0.11, *p* = 0.463) (Figure 6). There were significant differences (*p* < 0.05) in the sand and silt content of the two systems. However, silt was a redundant variable and was therefore removed from the RDA analysis. The RDA model was not statistically significant (*p* = 0.571), indicating no significant relationship between the measured soil variables and nematode community composition (Figure 7).

**TABLE 2:** Nematode‐based indices in organic and conventional spinach cropping systems in Embu, Kenya.

Index	Organic system		Conventional system		*p* *
	Mean	SE	Mean	SE	
Maturity Index	2.23	0.22	2.32	0.18	0.75
Maturity Index 2–5	2.59	0.09	2.52	0.1	0.60
Plant Parasitic Index	3.32	0.1	3.59	0.12	0.12
Channel Index	21.96	10.02	34.17	9.23	0.40
Basa Index	18.62	3.61	25.25	5.84	0.36
Enrichment Index	60.47	10.84	44.04	12.27	0.34
Structure Index	66.19	5.74	63.02	7.95	0.75

* Differences considered significant at *p* < 0.05.

## 4 Discussion

Intensive agriculture has led to a decline in soil health and sustainable agricultural practices are preferred (Squire et al. 2015). In the present study, 394 ASVs representing 73 nematode genera were detected across organic and conventional spinach fields. This is consistent with other studies in agricultural systems (Akanwari et al. 2025; Bell et al. 2021). In this study, no significant differences were observed in the relative abundance of nematode families, genera, or trophic groups between cropping systems. This is consistent with previous studies suggesting that management practices such as tillage, fertilization, irrigation, and pesticide application may exert a stronger influence on nematode assemblages than cropping system type alone (Bongiorno et al. 2019; Neher 1999; Rijssel et al. 2025). Conversely, other studies have documented greater abundance of Cephalobidae in conventional fields and attributed this to the fact that cp‐2 bacterial feeders can persist in systems with mineral fertilizers where nutrient inputs are largely inorganic and microbial communities are less diverse (Neher 1999). Other studies have reported a higher abundance of Cephalobidae in organic systems due to high levels of organic matter, N and C (Treonis et al. 2018). Similarly, Prismatolaimidae has been found in higher numbers in organic soils (Neher 1999; Quist et al. 2016) which differs from our observations. In addition, Neher (1999) reported a high abundance of Trichodoridae in organic soils possibly influenced by the type of host crop. Contrary to our results, a higher abundance of Rhabditidae was linked to organic soils due to the fact that the family comprises enrichment opportunists that respond rapidly to organic amendments such as compost and manure (Atandi et al. 2017; Bongers and Bongers 1998; Treonis et al. 2018). Overall, these contrasting findings show that nematode community responses are highly context dependent. They are influenced by differences in management practices and input regimes across studies. This highlights the need for further research to explain these inconsistent patterns.

According to our findings there were no significant differences in the relative abundance of trophic groups between the two systems; an observation that was also reported by Neher (1999). Other factors such as cropping practices may have a more critical role in shaping the nematode communities (García‐Álvarez et al. 2004). Other studies, however, have shown variations in different trophic groups. For instance, Bulluck et al. (2002) reported a higher abundance of fungivores in organically managed farms and also found that bacterivores dominated organic systems, a pattern later reported by Betancur‐Corredor et al. (2023). The dominance of these enrichment opportunists is attributed to the organic amendments promoting bacterial activity and favoring bacterial decomposition (Bulluck and Ristaino 2002; Ferris et al. 2004; Ferris and Matute 2003). Other studies have also reported high levels of omnivores and predators due to enhanced resource heterogeneity from organic inputs (Grabau et al. 2017), the increased microbial abundance and nematode densities (Betancur‐Corredor et al. 2023). In the present study, such trophic responses were not observed, possibly due to site‐specific factors influencing nematode communities under organic inputs.

In this study, the PPI and CI were numerically higher in the conventional system, while the BI, EI, and SI were lower compared to the organic system; however, none of these differences were statistically significant. This pattern of similarity in food web indices is consistent with Neher (1999), and indicates that both systems may exhibit comparable food web structure despite differing management practices. In addition, past management practices may shape nematode communities regardless of the cropping system (Ugarte et al. 2013). Yang et al. (2021) also observed similar BI, CI, EI, and SI in organic and conventional vegetable farms. However, other studies have reported differences in these indices. Farms under conventional management with application of fertilizers and pesticides were shown to have higher PPI due to the positive effect of these inputs on parasitic nematodes. This was also linked to the indirect effects on plants which make them more susceptible to attack by parasitic nematodes (Tsiafouli et al. 2006). Organically managed farms were reported to have higher SI and EI (Li et al. 2018; Liu et al. 2016). Elevated EI values are typically associated with systems receiving organic inputs that stimulate bacterial growth and favor enrichment of opportunistic nematodes (Ferris et al. 2001; Liang et al. 2009). On the other hand, a high SI reflects improved trophic complexity resulting from a higher number of omnivores and predators (Ferris et al. 2001).

We did not record differences in the alpha and beta diversity of nematode communities in the two systems. In addition, the RDA model showed no significant associations between the abundance of the top 20 genera and soil properties, consistent with the findings by Chen et al. (2012). This homogeneity in community composition was also reported by Rijssel et al. (2025), who suggested that it could be due to the minimal influence of the cropping system. Our results agree with the findings by Neher (1999), where some nematode diversity indices in organic and conventional systems were comparable. Conversely, there are studies that show differences in nematode diversity in organically and conventionally managed systems (Quist et al. 2016; Yang et al. 2021). The lack of differences observed in the present study may be attributed to unmeasured environmental and management factors such as soil pH, moisture, compaction, or pesticide legacy effects. Greater spatial replication in future studies may also improve the detection of subtle management effects that were not detected in the present study.

## 5 Conclusion

This study assessed nematode communities in organic and conventional spinach production systems in Embu County, Kenya. It examined taxonomic composition, trophic structure, alpha and beta diversity, and nematode‐based indices. No statistically significant differences were observed in nematode families, genera, or trophic groups, or in alpha and beta diversity between the two systems. Nematode‐based indices also showed no significant variation between organic and conventional farms. The results provide baseline information on nematode communities in spinach agroecosystems. The findings suggest that cropping system alone was not a strong determinant of nematode community variation in the study area. The effects of seasons, climate, and nutrient source on nematode diversity were not considered. Future studies can build on these findings by incorporating multi‐season sampling, climatic gradients, and different organic and inorganic inputs. Expanding spatial coverage to include other regions and soil types would also provide a broader perspective on how farming practices influence nematode communities.

## Funding

This work was supported by the Society for the Protection of Underground Networks.

## Conflicts of Interest

The authors declare no conflicts of interest.

## Supporting information


**Figure S1:** Rarefaction curves of samples from conventional and organic spinach farms in Embu, Kenya. Note: sequencing depth starts at zero.

## Data Availability

The sequencing data are available in the National Center for Biotechnology Information database under BioProject ID PRJNA1426156. On reasonable request, the corresponding authors will provide any other datasets analyzed during the current work.
