# Supplementary material for: Nematode Community Profiling in Organic and Conventional Spinach Systems
Source: Plant Environ Interact. 2026 Aug 4;7(4):e70201. doi: 10.1002/pei3.70201 (PMC13438177; doi:10.1002/pei3.70201)
Supplement: Supplementary file 1 [file PEI3-7-e70201-s001.docx]

**Nematode community profiling in organic and conventional spinach systems**

**Scolasticah K. Munene^a^**, **Hannah Karuri ^a*^**, **Mariciano Mutiga ^b^**

^a^ **Department of Biological Sciences, University of Embu, P.O Box 6-60100, Embu, Kenya**
^b^ **Department of Dryland Agriculture and Natural Resources Management, P.O Box 193-60215 Marimanti, Meru, Kenya**

**^*^Corresponding author email address:** [hwkaruri@gmail.com](mailto:hwkaruri@gmail.com)

**Figure S1: Rarefaction curves of samples from conventional and organic spinach farms in Embu, Kenya. Note: sequencing depth starts at zero.**
